# Enteral glutamine supplementation in critically ill patients: a systematic review and meta-analysis

**DOI:** 10.1186/s13054-015-1002-x

**Published:** 2015-08-18

**Authors:** Arthur R. H. van Zanten, Rupinder Dhaliwal, Dominique Garrel, Daren K. Heyland

**Affiliations:** Department of Intensive Care Medicine, Gelderse Vallei Hospital, Willy Brandtlaan 10, 6716 RP Ede, The Netherlands; Clinical Evaluation Research Unit, Kingston General Hospital, Angada 4, 76 Stuart Street, Kingston, ON K7L 2V7 Canada; Department of Nutrition, University of Montreal, Pavillon Liliane de Stewart, 2405, Chemin de la Côte-Sainte-Catherine, Local 1204, Montreal, QC H3T 1A8 Canada

## Abstract

**Introduction:**

Glutamine (GLN) has been suggested to have a beneficial influence on outcomes of critically ill patients. However, recent large-scale trials have suggested harm associated with GLN supplementation. Recently, systematic reviews on the use of parenteral GLN have been published; however, less information is available on the role of enteral GLN. Therefore, the aim of this systematic review was to study the effects of enteral GLN supplementation in patients with critical illness.

**Methods:**

We identified randomized controlled trials conducted from 1980 to 2014 with enterally administered GLN in adult critically ill patients. Studies of parenteral GLN only or combined enteral-parenteral GLN were excluded. The methodological quality of studies was scored, and trial data were statistically combined. We examined a priori the treatment effects in subgroups of trials of burn and trauma patients.

**Results:**

A total of 11 studies involving 1079 adult critically ill patients and enteral GLN supplementation were identified. Enteral GLN supplementation was not associated with a reduction of hospital mortality (risk ratio [RR] 0.94, 95 % confidence interval [CI] 0.65–1.36; *p* =0.74), infectious complications (RR 0.93, 95 % CI 0.79–1.10; *p* =0.39) or stay in the intensive care unit (weighted mean difference [WMD] −1.36 days, 95 % CI −5.51 to 2.78; *p* =0.52). However, there was a significant reduction in hospital stay (WMD 4.73 days, 95 % CI −8.53 to −0.90; *p* =0.02). In the subset of studies of patients with burns, enteral GLN supplementation was associated with significant reductions in hospital mortality (RR 0.19, 95 % 0.06–0.67; *p* =0.010) and hospital stay (WMD −9.16, 95 % CI −15.06 to −3.26; *p* =0.002). There was no effect in trauma patients.

**Conclusions:**

Enteral GLN supplementation does not confer significant clinical benefit in critically ill patients, with the exception of reduced hospital stay. There may be a significant benefit in patients with burns, but data are sparse and larger randomized trials are warranted to confirm this effect.

## Introduction

Immune-modulating nutrients are considered to modulate inflammatory and oxidative stress responses and to optimize the impaired (cellular) immune function [1]. Glutamine (GLN) is the most abundant free (non-essential) amino acid of the 20 amino acids in humans. No deficiencies are likely to be present in healthy persons, as GLN can be synthesized de novo. However, in catabolic and stress states that are commonly present in critically ill, trauma and burn patients, low plasma levels of GLN upon intensive care unit (ICU) admission have been encountered, leading to the assumption that these levels are too low for the actual medical condition and that GLN should be considered conditionally deficient [2]. The metabolic effects of GLN suggest a role in the inflammatory and oxidative stress responses [3]. On the basis of the association of low plasma GLN levels (<420 μmol/L) upon ICU admission and increased hospital mortality, one study group suggested that GLN supplementation in critically ill patients could be essential [4].

Since then, many studies on parenteral and/or enteral GLN supplementation in critically ill patients have been performed, with the earliest of these published in 1997 [5]. The authors of several older systematic reviews and meta-analyses reported that GLN supplementation, combined with enteral nutrition (EN) and parenteral nutrition (PN), is associated with reduced infectious morbidity and improved recovery from critical illness compared with standard nutrition [6–8].

Results of earlier meta-analyses were based mainly on small, single-center studies. This led to the development of international guidelines for the use of enteral GLN in critically ill patients [9–11].

However, in the latest meta-analysis published by the Cochrane Database of Systematic Reviews, signals for mortality reduction were lost and only moderate- and low-level evidence on reduction of morbidity was found, with high risk of overall bias, suspected publication bias and moderate to substantial heterogeneity within the included studies [12].

Most recently, two large, multicenter studies—the Reducing Deaths Due to Oxidative Stress (REDOXS) and MetaPlus trials—have shown no effects of GLN on infectious morbidity; however, more importantly, increased long-term mortality in critically ill patients in the GLN supplementation arms was demonstrated [13, 14]. Together, the results of these two studies challenge current guidelines and recommendations for enteral and/or parenteral GLN in critically ill patients, as safety concerns have been communicated [15].

An up-to-date review on parenteral use of GLN has become available recently [16]; however, no systematic analysis focused on enteral GLN supplementation in critical illness has been performed over the last 6 years [17]. Moreover, a similar dose of GLN or glutamine-alanine dipeptide administered through the enteral versus the parenteral route has smaller effects on plasma GLN levels, possibly owing to splanchnic extraction [18]. Furthermore, investigators in many trials on PN supplemented with GLN (typically without GLN in the control product) have studied patients not receiving EN. In contrast, in the control groups in the EN GLN studies, the standard commercially available EN typically contains limited amounts of GLN (5–6 g/L). Therefore, available data on parenteral GLN supplementation cannot be extrapolated to EN supplementation and thus may not be used as a basis for recommendations for enteral GLN administration.

In the present systematic literature review and meta-analysis, we address the question whether enteral administration of GLN as part of nutrition support has a positive effect on clinical outcomes in general, trauma and burn injury patients who were critically ill.

## Methods

### Study identification

The following databases were searched for articles published between 1980 and September 2014: Embase, MEDLINE, CINAHL, the Cochrane Central Register of Controlled Trials and the Cochrane Database of Systematic Reviews. In the literature search, we used broad search terms containing “randomized,” “blind,” “clinical trial,” “nutrition,” “nutritional support” or “dietary supplementation” or “enteral nutrition” or “parenteral nutrition” or “parenteral nutrition solutions” and “critical care” or “critical illness” or “intensive care unit”. The results were then reviewed to identify articles describing enteral GLN supplementation. A unique feature of this meta-analysis is that no language restrictions were placed on the searches. The authors personal files and reference lists of relevant review articles were also reviewed. As this is a systematic review, no ethics board approval or patient consent was required.

### Study selection criteria

We included original studies only if they met the following inclusion criteria:

1. *Study design*: randomized clinical trials.
2. *Study population*: critically ill adult patients (>18 years of age), defined as patients admitted to an ICU. When this information was unclear, we considered a mortality rate higher than 5 % (hospital mortality or, if this was not reported, ICU mortality or 28-day mortality) in the control group to be consistent with critical illness.
3. *Intervention*: enteral GLN versus control (isonotrogenous control).
4. *Study outcomes*: must have included one of the following: mortality, ICU and hospital lengths of stay (LOSs) and infectious complications.

Studies in which the authors reported only other clinical endpoints, such as duration of mechanical ventilation, and studies of parenteral GLN only or combined enteral and parenteral GLN were excluded.

### Data abstraction

Decisions about the inclusion of the articles were made in duplicate. Two reviewers, using a data abstraction form with a scoring system, reviewed all original studies independently. An assessment of the criteria for inclusion, details on the patient population, intervention and control and/or placebo, and clinical outcomes was done as described in earlier publications [19]. Using a scoring system we previously developed, we assessed the methodological quality of individual trials according to (1) whether randomization was concealed, (2) blinding, (3) whether the analysis was based on the intention-to-treat principle, (4) patient selection, (5) comparability of groups at baseline, (6) extent of follow-up, (7) description of treatment protocol and cointerventions and (8) definition of clinical outcomes [19]. Each individual study was scored from 1 to 14 (Table 1). Disagreement regarding the individual scores of each of the categories was resolved by consensus between the two reviewers. We attempted to contact the authors of included studies and requested additional information not contained in their published articles.

**Table 1:** Study scoring data abstraction form used to score all original studies independently

	Score		
	0	1	2
Randomization	–	Not concealed or not sure	Concealed randomization
Analysis	Other	–	Intention to treat
Blinding	Not blinded	Single-blind	Double-blind
Patient selection	Selected patients or unable to tell	Consecutive eligible patients	–
Comparability of groups at baseline	No or not sure	Yes	–
Extent of follow-up	<100 %	100 %	–
Treatment protocol	Poorly described	Reproducibly described	–
Cointerventions	Not described	Described but not equal or not sure	Well described and all equal
Outcomes	Not described	Partially described	Objectively defined

### Data synthesis

The primary outcome of the systematic review was hospital mortality. From all studies, we extracted data regarding hospital mortality if reported (specified or assumed to be hospital mortality if not specified). If hospital mortality was not reported, we used ICU mortality or 28-day mortality. Secondary outcomes included infection and ICU and hospital LOSs. We used definitions of infections as defined by the authors of the original articles. We combined data from all trials to estimate the pooled risk ratio (RR) with 95 % confidence interval (CI) for mortality and infectious complications and overall weighted mean difference (WMD) with 95 % CI for LOS data. Pooled RRs were calculated using the Mantel-Haenszel test, and WMDs were estimated using the inverse variance approach. The random-effects model of DerSimonian and Laird was used to estimate variances for the Mantel-Haenszel and inverse variance estimations [20]. All analyses except the test for asymmetry were conducted using Review Manager (RevMan) 5.1 software [21].

When possible, studies were aggregated on an intention-to-treat basis (Table 2). The presence of heterogeneity was tested using a weighted Mantel-Haenszel *χ*^2^ test and quantified by the *I*^2^ statistic as implemented in RevMan 5.1 [21, 22]. Upon review of the dataset, we found that one randomized controlled trial contained other supplemental nutrients, not just GLN. To evaluate the effect of that trial on the overall results, we performed a sensitivity analysis wherein we excluded the trial to see how it affected the overall results [14]. The possibility of publication bias was assessed by generating funnel plots and testing asymmetry of outcomes using methods proposed by Rucker and colleagues [23]. We considered *p* < 0.05 to be statistically significant and *p* < 0.20 as the indicator of trend.

**Table 2:** Included randomized studies of enteral glutamine supplementation in critically ill patients

Author	Year	ICU population	Setting	All patients	GLN+ patients	GLN− patients	Reference
Houdijk et al.	1998	Critically ill trauma (100 %)	Single center	80	41	39	[24]
Jones et al.	1999	Mixed ICU (6 burns, 6 trauma)	Single center	50	26	24	[25]
Brantley and Pierce	2000	Critically ill trauma (100 %)	Single center	72	31	41	[26]
Hall et al.	2003	Mixed ICU (mostly trauma, 7 burn)	Single center	363	179	184	[27]
Garrel et al.	2003	Burns (TBSA: 20–80 %)	Single center	45	21	24	[28]
Zhou et al.	2003	Severe burns TBSA 50–80 %	Single center	40	20	20	[29]
Peng et al.	2004	Severe burns TBSA >30 %	Single center	48	25	23	[30]
Luo et al.	2008	Mixed ICU Medical-surgical	Single center	30	15	15	[31]
McQuiggan et al.	2008	Shock trauma patients	Single center	20	10	10	[32]
Pattanshetti et al.	2009	Burns (TBSA: 20–60 %)	Single center	30	15	15	[33]
van Zanten et al.	2014	Mixed ICU (109 trauma)	Multicenter	301	152	149	[14]

*GLN+* patients treated with glutamine supplemented enteral nutrition, *GLN−* patients treated with control enteral nutrition, *ICU* intensive care unit, *TBSA* total body surface area

### Subgroup analyses

We performed a predefined subgroup analysis to assess a number of possible influences on the effect of enteral GLN supplementation on clinical outcomes. We first explored whether there was a different treatment effect of enteral GLN in patients with burn injury and patients with trauma. The trial done by van Zanten and colleagues also contained an a priori subgroup analysis of patients with trauma, and we were able to obtain the data for the subset of trauma patients and include these data in the subgroup analysis [14]. We also assessed the effect of trial quality on outcome, as it is often hypothesized that, compared with trials of higher methodological quality, trials of lower methodological quality tend to yield more positive clinical signals for the therapy being tested. Using our trial scoring tool, we designated trials with a methodological score of 9 (out of a maximum score of 14) or more (median of scoring of all trials) as a high-quality trial for the purposes of this review.

## Results

### Study identification and selection

The literature search yielded 42 potentially eligible randomized controlled trials, of which 11 with a total of 1079 patients were included in our systematic review (see Table 2) [14, 24–33]. In total, 535 patients were treated with GLN supplementation and 544 patients with a control feed.

As shown in Table 3, a total of 33 studies [34–65] were excluded for the following main reasons: (1) patients not considered to be adult critically ill patients (n =9); (2) no clinical outcomes meeting inclusion criteria (n =9); (3) being duplicate studies, reviews of published trials or subgroups of included studies (n =6); (4) crossover study design (n =4); and/or (5) multiple other interventions were studied, such as arginine, glycine, probiotics and fibers (n =4).

**Table 3:** Excluded randomized studies of enteral glutamine supplementation in critically ill patients

Author	Year	Reasons for exclusion	References
Jebb et al.	1995	Transplant and/or elective surgery patients	[34]
Long et al.	1995	No clinical outcomes	[35]
Jensen et al.	1996	No clinical outcomes	[36]
Fish et al.	1997	Cancer patients	[37]
Scolapio et al.	1997	Crossover design	[38]
Anderson et al.	1998	Surgical patients	[39]
Anderson et al.	1998	Pediatric cancer patients	[40]
Den Hond et al.	1999	Not ICU patients	[41]
Schloerb and Skikne	1999	Cancer and/or surgery patients	[42]
Scolapio	1999	Crossover design	[43]
Zhou et al.	1999	Earlier study of 2003 RCT already included	[44]
Jackson et al.	2000	Surgery patients, no clinical outcomes	[45]
Szkudlarek et al.	2000	Crossover design	[46]
Chen et al.	2001	No clinical outcomes	[47]
Scolapio et al.	2001	Crossover design	[48]
Velasco et al.	2001	No clinical outcomes, duplicate of Houdijk et al. study [24]	[49]
Boelens et al.	2002	No clinical outcomes	[50]
Novak et al.	2002	Studies on critically ill patients were included in this review	[51]
Flaring et al.	2003	Elective surgery patients	[52]
García-de-Lorenzo et al.	2003	Systematic review, Individual studies were included in this review	[53]
Boelens et al.	2004	Duplicate of Houdijk et al. study [24]	[54]
Falcao de Arruda et al.	2004	Includes probiotics	[55]
Peng et al.	2005	Duplicate study of earlier publication already [30] included	[56]
Peng et al.	2006	Duplicate of a previous study [30]	[57]
Guo et al.	2007	No clinical outcomes	[58]
Kuhls et al.	2007	Too many interventions	[59]
Spindler-Vesel et al.	2007	Too many interventions: RCT of GLN vs. fiber vs. peptide vs. fiber + synbiotics	[60]
Beale et al.	2008	Non-isonitrogenous intervention including arginine and glycine	[61]
Han et al.	2012	Elective surgery patients	[62]
Cavalcante et al.	2012	No clinical outcomes, crossover design	[63]
Han et al.	2014	No clinical outcomes	[64]
Koksal et al.	2014	Only duration of mechanical ventilation reported	[65]

*GLN* glutamine, *ICU* intensive care unit, *RCT* randomized controlled trial

Thus, we ultimately included 11 studies of enteral GLN supplementation performed in ICU patients with diagnoses ranging from trauma to burns and sepsis, as described in Table 3 [14, 24–33]. The results were based on data derived from the included studies, depicted in Table 4.

**Table 4:** Relevant outcome parameters of included randomized studies of enteral glutamine supplementation in critically ill patients

Study	Methods	Intervention	Mortality, n (%)^a^		Infections, n (%)^b^		Hospital stay (days)		ICU LOS (days)	
	Score	Dose (g/kg/day)	Experimental	Control	Experimental	Control	Experimental	Control	Experimental	Control
		Type of feeding								
Houdijk et al. [24]	C. random: Yes	>0.25	4/41 (9.8)	3/39 (7.7)	20/35 (57.1)	26/37 (70.2)	32.7 ± 17.1	33.0 ± 23.8	NA	NA
	ITT: No	Altira Q (glutamine-enriched formula) vs. isonitrogenous control (added amino acids)								
	Blinding: Yes									
	10									
		Same volume of feeding received in both groups								
Jones et al. [25]	C. random: Yes	0.16	Hospital	Hospital	NA	NA	NA	NA	11 (4–54)	16.5 (5–66)
	ITT: No	Protina Torre MP (Fresenius Kabi, Bad Homburg, Germany) + glutamine (10–15 g/day nitrogen) vs. isonitrogenous control (11–14 g/day nitrogen)	10/26 (38.5)	9/24 (37.5)						
	Blinding: Yes		ICU	ICU						
	8		9/26 (35)	9/24 (38)						
			6 months	6 months						
			12/26 (46)	10/24 (42)						
Brantley and Pierce [26]	C. random: Not sure	0.50	0/31 (0.0)	0/41 (0.0)	NA	NA	19.5 ± 8.8	20.8 ± 11.5	11.4	11.1
	ITT: No	Glutamine-supplemented enteral formula vs. standard formula (isonitrogenous) protein given 1.5 g/kg/day								
	Blinding: No									
	4									
Hall et al. [27]	C. random: Yes	0.27	Hospital	Hospital	38/179 (21)	43/184 (23)	25 (16–42)^c^	30 (19–45)^c^	11 (7–19) (excluding deaths)	13 (8–19) (excluding deaths)
	ITT: Yes	Isocal (Nestlé Health Science, Lutry, Switzerland) + glutamine (66 g/day protein) vs. isonitrogenous formula Isocal + glycine (64 g/day protein)	24/179 (13)	23/184 (13)						
	Blinding: Yes		ICU	ICU						
	13		16/179 (9)	14/184 (8)						
			30 days	30 days						
			26/179 (15)	25/184 (14)						
			6 months	6 months						
			27/179 (15)	30/184 (16)						
Hall et al. [27]	C. random: Yes	0.27	7/76 (9)	6/78 (8)	Sepsis	Sepsis	NA	NA	NA	NA
Trauma subgroup	ITT: Yes	Isocal + glutamine (66 g/day protein) vs. isonitrogenous formula Isocal + glycine (64 g/day protein)			7/76 (9)	11/78 (14)				
	Blinding: Yes									
	13									
Garrel et al. [28]	C. random: Yes	0.28	2/21 (10)	12/24 (50)	Positive blood cultures	Positive blood cultures	33 ± 17 (16)^d^	29 ± 17 (19)^d^	NA	NA
	ITT: yes	Sandosource (Nestlé Health Science) + glutamine (2.15 g/kg/day protein) vs. Sandosource + amino acids (isonitrogenous), 1.97 g/kg/day protein			7/19 (37)	10/22 (45)				
	Blinding: Yes									
	11									
Zhou et al. [29]	C. random: Yes	0.35	0/20	0/20	2/20 (10)	6/20 (30)	67 ± 4 (20)	73 ± 6 (20)	NA	NA
	ITT: No	Ensure (NutriDrinks, Perivale, UK) + glutamine vs. Ensure + amino acids (isonitrogenous)								
	Blinding: Double-blind									
	8									
Peng et al. [30]	C. random: Not sure	0.5	NA	NA	NA	NA	46.6 ± 12.9 (25)	55.7 ± 17.4 (23)	NA	NA
	ITT: Yes	Oral glutamine granules vs. placebo (isocaloric, isonitrogenous) 2.0 g/kg/day protein								
	Blinding: No									
	7									
Luo et al.^e^ [31]	C. random: Not sure	0.32	ICU	ICU	NA	NA	NA	NA	8.1 ± 0.4 (12)	6.9 ± 0.9 (9)
	ITT: No	Glutamine + IV saline + vs. Nutren (Nestlé Health Science) + 15 % Clinisol (Baxter Healthcare, Deerfield, IL, USA) (placebo) (isocaloric, isonitrogenous)	1/12	0 /9						
	Blinding: Double-blind		28 days	28 days						
	9	1.7 g/kg/d protein	1/12	0 /9						
McQuiggan et al. [32]	C. random: Not sure	0.5 (actual 0.4) IMPACT (Nestlé Health Science) + Glutasolve (Nestlé Health Science) via NJ tube (1.3 g/kg/day protein), bolus with H_2_O vs. Impact + protein supplements (isonitrogenous, isocaloric) 0.85 g/kg/day protein	0/10	2/10 (20)	NA	NA	32 ± 13.6 (10)	39.3 ± 33.6 (10)	4.8 ± 6.7 (10)	10.4 ± 6.2 (10)
	ITT: Yes									
	Blinding: No									
	10									
Pattanshetti et al. [33]	C. random: Not sure	Enteral isonitrogenous mixture + EN glutamine + “regular” nutrition vs. enteral isonitrogenous mixture + “regular” nutrition	0/15	2/15	Number of times positive blood cultures	Number of times positive blood cultures	22.73 ± 9.13	39.73 ± 18.27	NA	NA
	ITT: Yes									
	Blinding: Single-blind (outcomes)									
	8				0.20 ± 0.41	0.73 ± 0.96				
van Zanten et al. [14]	C. random: Yes	0.28 (mean intake) glutamine, omega-3, antioxidant-enriched EN (experimental product) vs. isonitrogenous, isocaloric high-protein EN (Nutrison Advanced Protison; Nutricia Advanced Medical Nutrition, Amsterdam, the Netherlands)	Hospital	Hospital	80/152 (53)	78/149 (52)	38.2 ± 28.9	37.7 ± 27.5	23.7 ± 22.4	25.6 ± 24.0
	ITT: Yes		38/152 (25)	33/149 (22)						
	Blinding: Double-blind		ICU	ICU						
	12		30/152 (20)	29/149 (20)						
			28 days	28 days						
			31/152 (20)	25/149 (17)						
			6 months	6 months						
			53/152 (35)	42/149 (29)						
van Zanten et al. [14] trauma subgroup	C. random: Yes	0.28 (mean intake) glutamine, omega-3, antioxidant-enriched EN (experimental product) vs. isonitrogenous, isocaloric high-protein EN (Nutrison Advanced Protison)	Hospital	Hospital	32/55 (58)	36/54 (67)	44.4 ± 31.2	39.8 ± 25.3	31.3 ± 30.3	32.5 ± 27.5
	ITT: Yes		6/55 (11)	6/54 (11)						
	Blinding: Double-blind		ICU	ICU						
	12		5/55 (9)	6/54 (11)						
			28 days	28 days						
			4/55 (7)	2/54 (4)						
			6 months	6 months						
			8/55 (15)	59/54 (17)						

*C. random* concealed randomization median (range), *EN* enteral nutrition, *ITT* intent to treat, *IV* intravenous, *NA* not applicable, *NJ* nasojejunal, *TPN* total parenteral nutrition

Data are presented as mean ± standard deviation or number (%), as appropriate

^a^Hospital mortality unless otherwise stated

^b^Number of patients with infections unless otherwise stated

^c^Median and range hence not included in meta-analysis (Hall et al. 2003 [27]; *p* = not significant)

^d^Subgroup of patients, hence not included in the meta-analyses [28]

^e^Data from parenteral glutamine group not shown here

### Effect of enteral glutamine supplementation on hospital mortality

**Fig. 1:**
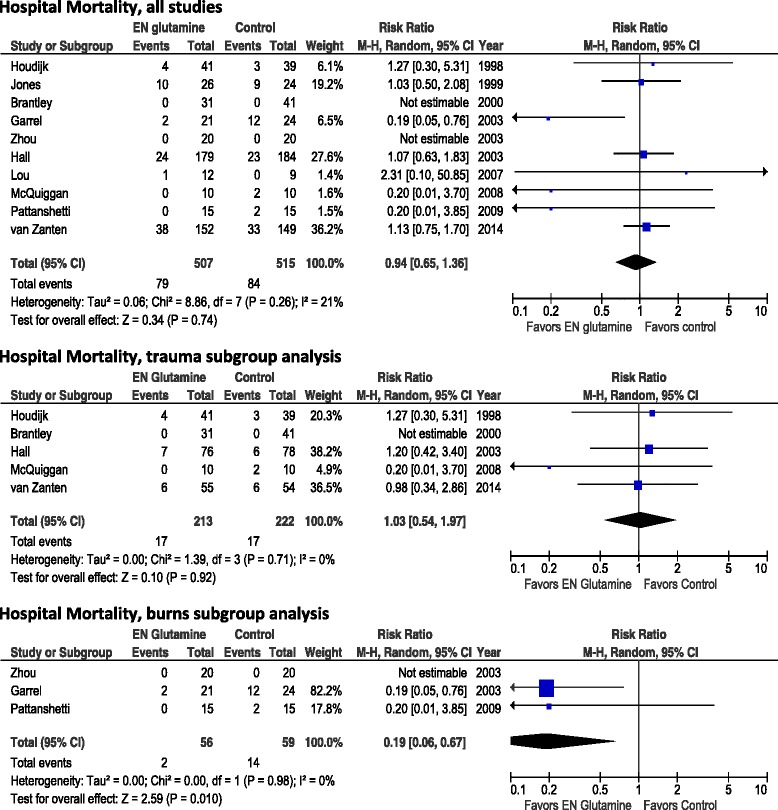
Effects of enteral glutamine on hospital mortality. *95 % CI* 95 % confidence interval, *EN* enteral nutrition, *M-H* Mantel-Haenszel

When the data from 10 of the 11 total identified EN GLN studies that reported on mortality (Fig. 1) were aggregated, enteral GLN supplementation was not associated with a reduction in hospital mortality (RR 0.94, 95 % CI 0.65–1.36; *p* =0.74; test for heterogeneity *I*^2^ = 21 %). The combined hospital mortality was 79 (15.6 %) of 507 in the GLN group and 84 (16.3 %) of 515 in the control group. In the sensitivity analysis without the van Zanten et al. trial [14], there was still no effect on mortality (RR 0.80, 95 % CI 0.46–1.38; *p* =0.42; heterogeneity *I*^2^ = 27 %). Also, in the subgroup of trauma patients, no effect on hospital mortality was found (RR 1.03, 95 % CI 0.54–1.97; *p* =0.92; heterogeneity *I*^2^ = 0 %; n =5 studies). However, in the small subgroup of burn patients, a statistically significant reduction in mortality (2 [3.6 %] of 56 versus 14 [23.7 %] of 59) was demonstrated (RR 0.19, 95 % CI 0.06–0.67; *p* =0.010; heterogeneity *I*^2^ = 0 %; n =3 studies).

### Effect of glutamine supplementation on infectious complications

**Fig. 2:**
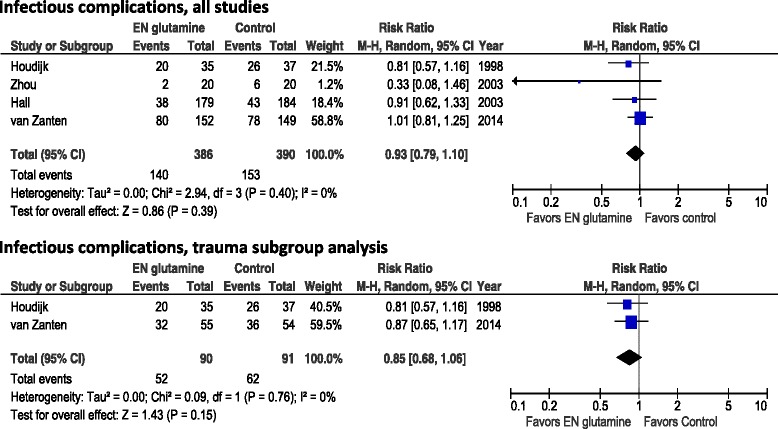
Effects of enteral glutamine on infectious complications. *95 % CI* 95 % confidence interval, *EN* enteral nutrition, *M-H* Mantel-Haenszel

When the four studies in which the researchers reported infectious complications were aggregated, enteral GLN supplementation was not associated with a reduction in infectious complications (RR 0.93, 95 % CI 0.79–1.10, *p* =0.39; heterogeneity *I*^2^ = 0 %) (Fig. 2). The overall incidence of infection was 140 (36.3 %) of 386 in the GLN group and 153 (39.2 %) of 390 in the control group. A sensitivity analysis without the van Zanten et al. study [14] showed a trend toward a reduction in infectious morbidity (RR 0.83, 95 % CI 0.64–1.08, *p* =0.16; heterogeneity *I*^2^ = 0 %). Also, in the subgroup of trauma patients, a trend toward a reduction in infectious morbidity was found (RR 0.85, 95 % CI 0.68–1.06, *p* =0.15; heterogeneity *I*^2^ = 0 %; n =2 studies). In the small subgroup of burn patients, few data on infections were available. Zhou et al. [29] reported infections in 2 (10 %) of 20 of burn patients treated with GLN versus 6 (30 %) of 20 in the control group. Garrel et al. [28] showed reductions in positive blood cultures in 7 (37 %) of 19 in GLN-treated patients versus 10 (45 %) of 22 in control subjects.

### Effect of glutamine supplementation on ICU length of stay

**Fig. 3:**
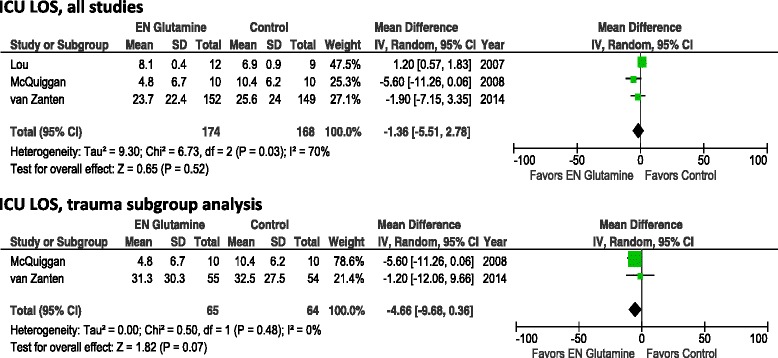
Effects of enteral glutamine on ICU length of stay. *95 % CI* 95 % confidence interval, *EN* enteral nutrition, *ICU* intensive care unit, *IV* intravenous, *LOS*, length of stay, *SD* standard deviation

When we aggregated data from the three studies in which authors reported ICU LOS as mean ± standard deviation (Fig. 3), we found that enteral GLN supplementation was not associated with a reduction in ICU LOS (WMD −1.36, 95 % CI −5.51 to 2.78; *p* =0.52; heterogeneity *I*^2^ = 70 %). When we excluded the van Zanten et al. study [14], we also observed no effect on ICU LOS (WMD −1.59, 95 % CI −8.15 to 4.96; *p* =0.63; heterogeneity *I*^2^ = 82 %). In the small subgroup of trauma patients, we found a trend toward reduction in ICU LOS (WMD −4.66, 95 % CI −9.68 to 0.36; *p* =0.07; heterogeneity *I*^2^ = 82 %; n =2 studies). In the small number of trials with burn patients, no data on ICU LOS were available.

### Effect of glutamine supplementation on hospital length of stay

**Fig. 4:**
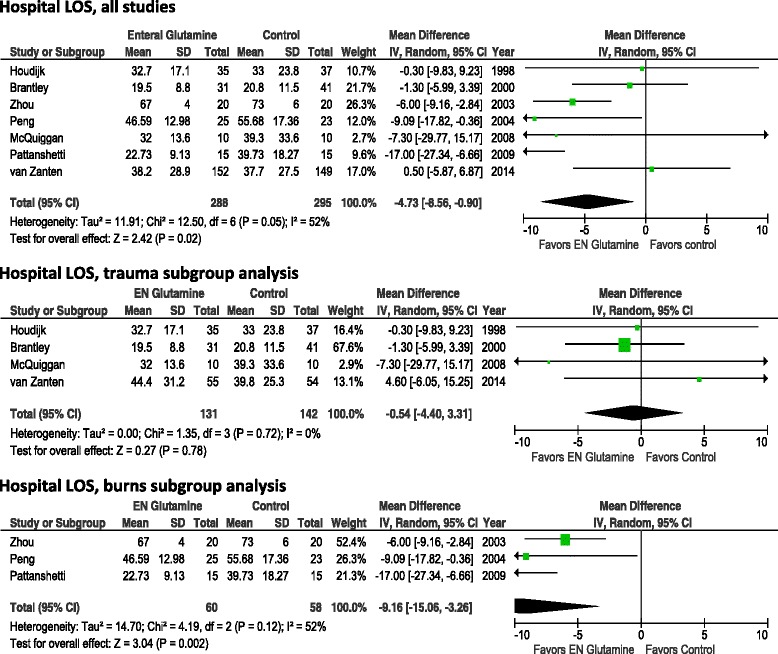
Effects of enteral glutamine on hospital length of stay. *95 % CI* 95 % confidence interval, *EN* enteral nutrition, *IV* intravenous, *LOS*, length of stay, *SD* standard deviation

When we aggregated the seven studies in which investigators reported data on hospital LOS (Fig. 4), we found that GLN supplementation was associated with a significant reduction in hospital LOS (WMD −4.73, 95 % CI −8.56 to −0.90; *p* =0.02; heterogeneity *I*^2^ = 52 %). The finding of a significant reduction in hospital LOS persisted after we excluded the van Zanten et al. study [14] (WMD 6.95 days, 95 % CI −12.37 to −1.53; *p* =0.01; heterogeneity *I*^2^ = 76 %). In the subgroup of trauma patients, no reduction in hospital LOS was found (WMD −0.54, 95 % CI −4.40 to 3.31, *p* =0.78; heterogeneity *I*^2^ = 0 %; n =4 studies). In the small subgroup of burn patients, we found a significant reduction in hospital LOS (WMD −9.16, 95 % CI −15.06 to −3.26; *p* =0.002; heterogeneity *I*^2^ = 52 %; n =3 studies).

### Effect of study quality on outcomes

There was no effect of enteral GLN on reduction in hospital mortality in high-quality trials (RR 0.90, 95 % CI 0.55–1.48; *p* =0.69) compared with low-quality trials (RR 0.84, 95 % CI 0.28–2.50; *p* =0.75) when we tested for subgroup differences (*p* =0.90; data not shown). There was an insufficient number of trials in which authors reported data on infectious outcomes and LOS in the low- and high-quality trial categories to allow for these comparisons to be made.

### Risk of publication bias across studies

Funnel plots for all outcomes were created to assess for publication bias (data not shown). The test of asymmetry was not found to be significant for any of the endpoints, including hospital mortality (*p* =0.18), infectious complications (*p* =0.23), ICU LOS (*p* =0.25) and hospital LOS (*p* =0.48).

## Discussion

Our overall results reveal that enteral GLN does not confer reductions in hospital mortality, with the exception of burn patients. We observed marked heterogeneity among the included studies, which are described in detail in Table 4. In our present analysis, we could not find strong signals of publication bias effects on the primary outcome parameter, hospital mortality. Whether this means that enteral GLN supplementation is safe for critically ill patients should be interpreted with caution, as previous analyses of parenteral GLN supplementation have demonstrated divergent effects on mortality when single-center studies were compared with multicenter trials; in other words, the observed beneficial effects on mortality were due to effects in single-center studies [16, 66]. With respect to enteral GLN, our analysis included only one multicenter study, and the results of that study suggest increased harm [14].

### Lack of effect on infectious morbidity

In contrast to earlier observations, we could not demonstrate any beneficial effect of enteral GLN on infectious morbidity. Only in burn patients was a small effect seen; however, the number of patients is limited, which precludes making strong recommendations. Recently, a lack of effect of GLN administration to boost the innate immune system response in trauma patients in the ICU has been demonstrated. No increase in the expression and/or functionality of Toll-like receptors, key receptors that sense infections, was found in response to GLN supplementation [67]. Furthermore, protein intake and infectious morbidity seem to be associated [68]. Therefore, the effects of GLN supplementation can be studied adequately only in isonitrogenous intervention studies.

### Reduction of hospital length of stay

Although a reduction in ICU LOS could not be demonstrated, a reduction in hospital LOS of approximately 4.7 days (WMD −4.73, 95 % CI −8.56 to −0.90) persisted. These results are in line with previously reported meta-analyses [17, 53]. This signal is driven largely by three studies of burn patients that, when aggregated, show a reduction of more than 9 days in the hospital.

### Glutamine in burn patients

Burn patients may be a unique group of patients for which GLN has clinically significant beneficial effects. Low blood GLN levels have been observed in this patient population [28, 29], and the conditionally essential hypothesis may apply to them. As noted in this review, several small trials have shown benefits with regard to blood infections [28] and LOS [29]. A similar effect was observed on Gram-negative blood infections in two studies with different routes of GLN administration [28, 69]. In a more recent study [33], researchers reported a decrease in blood infection of identical magnitude. Taken together, these observations strongly suggest that GLN has a significant effect on blood infections in burn patients. Decreased mortality was also found in one study with enteral GLN administration [28], although that trial had a high mortality rate in the control group. Although this study was not powered for testing an effect on mortality, the effect size observed warrants a larger trial. Such a large-scale, multicenter trial is currently underway, and stronger inferences about the use of GLN in burn patients awaits the results [70].

### Previous meta-analyses

In two meta-analyses of the initial randomized controlled trials of enteral GLN supplementation, García-de-Lorenzo et al. [53] and Jiang et al. [17] reported data on 17 and 7 studies, respectively. Although in the first meta-analysis the number of studies that included various patient categories was larger than in our present analysis, the number of studies addressing the effects of enteral GLN in critically ill patients was much lower. On the basis of their results, although the doses given and the duration of therapy varied widely depending on the pathologic condition, García-de-Lorenzo et al. [53] recommended using GLN intake of 20–30 g/day, early initiation of diet and maintenance for at least 5 days (grade C recommendations) [53]. Jiang et al. [17] concluded that administration of GLN-enhanced EN in patients with critical illness may reduce nosocomial infection rates and shorten hospital LOS. Furthermore, they recommended that studies be done with a large sample size to verify the efficiency of GLN-enhanced EN on lowering mortality in patients with critical illness. Since the publication of the García-de-Lorenzo et al. and Jiang et al. studies, another four studies have been published, including the multinational, multicenter MetaPlus study [14]. Incorporating data of these studies enabled us to evaluate GLN in more than 1000 patients.

### Safety concerns regarding glutamine supplementation

Two large multicenter trials, the REDOXS and the MetaPlus trials [13, 14], have fueled the debate on the safety and efficacy of GLN supplementation. Therefore, we believe they need to be discussed in more detail. The REDOXS trial was not included in our present analysis for reasons of both enteral and parenteral GLN supplementation [13].

The REDOXS trial was a factorial 2 × 2 randomized trial conducted in 40 ICUs in North America and Europe. A total of 1223 mechanically ventilated adult patients with multiple organ dysfunction syndrome were randomized to receive high doses of GLN, antioxidants, both or placebo separate from artificial nutrition [13]. Total caloric intake in both groups was about 900 kcal/day. The primary analysis demonstrated no clinical benefit and identified a trend toward increased mortality at 28 days (32.4 % vs. 27.2 %; adjusted odds ratio 1.28; 95 % CI 1.00–1.64; *p* =0.049) and a significant increase in hospital and 6-month mortality among patients who received GLN. There was no effect of antioxidants on 28-day mortality [13].

The MetaPlus trial was conducted from February 2010 through April 2012. It included a 6-month follow-up period in 14 ICUs in the Netherlands, Germany, France and Belgium. A total of 301 adult patients who were expected to be ventilated and to require EN for more than 72 h were randomized to the intervention feed (enriched with GLN and with antioxidants including selenium and fish oils) or control feed within 48 h of ICU admission and continued during ICU stay [14]. Consistent with attempting to supplement patients because of presumed nutrient deficiency, per 1500 ml, the enriched diet contained 113 g of protein, of which 23 g were alanyl-glutamine, and a total GLN content of 30 g; relatively high amounts of antioxidants, including 285 μg of selenium and an additional 7.5 g of fish oils. The control group received an isocaloric standard high-protein EN diet with similar amounts of proteins. There were no statistically significant differences in new infections according to the Centers for Disease Control and Prevention (CDC) definitions: 53 % (95 % CI 44–61 %) in the enriched group versus 52 % (95 % CI 44–61 %) in the control group (*p* =0.96). The study was designed to observe a 50 % relative reduction in incidence of new infections based on an incidence of 25 % in the control arm (absolute reduction of 12.5 %). The actual incidence of infections was larger than estimated (53 % and 52 %). Therefore, the study was not underpowered to detect differences in infections. Although the incidence of infections was higher than estimated, no reduction of infectious morbidity was observed. Secondary endpoints included mortality, Sequential Organ Failure Assessment scores, mechanical ventilation duration, ICU and hospital LOS and subtypes of infections according to CDC definitions. No differences were observed in secondary endpoints, except for a higher 6-month adjusted mortality rate in the enriched group (hazard ratio 1.57, 95 % CI 1.03–2.39; *p* =0.04) and an unadjusted higher mortality of 54 % in the medical subgroup (95 % CI 40–67 %) versus 35 % (95 % CI 22–49 %) (*p* =0.04). Mortality was a secondary endpoint in this study. However, we cannot ignore the observation of increased mortality just because it was a secondary endpoint. We have to look at these secondary endpoints when considering the safety of the intervention [71].

This meta-analysis does not suggest increased mortality with the use of enteral GLN supplementation. The signals of harm in the REDOXS trial may be due to the high dose of both enteral and parenteral GLN used, the negative effects in patients with renal failure and the low total caloric and protein intake, although these factors remain speculative. In the MetaPlus study, harmful effects were observed in all patients with respect to the adjusted 6-month mortality and unadjusted in the medical subgroup. The underlying mechanisms are still unclear, but data suggest that patients did worse if their baseline GLN plasma levels were higher. Another explanation could be that effects are due to the other immune-modulating ingredients or to an interaction among those.

### Is the glutamine conditional deficiency hypothesis still valid?

Overall, the benefits of enteral GLN supplementation seem to be limited. This should lead to a reevaluation of the importance and validity of the conditional deficiency hypothesis of GLN in critically ill patients. Some have suggested that low GLN plasma levels at ICU admission may be an adaptive response and that supplementation could be considered as a maladaptive response to this [72].

Several observations challenging the hypothesis have been published recently. The frequency of patients with low baseline plasma GLN levels is extremely variable and is not consistent [31, 73, 74]. There is no association of baseline plasma GLN and Acute Physiology and Chronic Health Evaluation II score, as could be expected when the severity of illness plays a role in conditional deficiency [74]. Moreover, in general and septic ICU patients, high baseline plasma GLN (>930 μmol/L) was associated with increased mortality suggesting a U-shaped association [74]. In addition, low baseline GLN levels were not always associated with increased mortality [73].

Considering conditional deficiency, the GLN rate of appearance from muscles to plasma is expected to be around the maximum production rate, estimated by isotopic techniques at 40–80 g/24 h [75]. However, this maximum muscle output could not be confirmed in a tracer study in ICU patients. The endogenous production of GLN in muscles and appearance in plasma were related to severity of illness and did not diminish by supplementation of GLN [76]. A trend toward higher mortality was demonstrated in patients with higher baseline GLN levels treated with GLN-enriched EN [14]. After ICU discharge, patients showed normalized plasma GLN levels not associated with long-term outcome. However, patients with the highest plasma GLN on the ICU discharge day showed a higher 1-year mortality [77].

### Consequences of findings

Our observations do not support use of GLN in critically ill patients; therefore, our present systematic review and meta-analysis is important. Moreover, the recent large-scale, multicenter trials (REDOXS and MetaPlus) show no benefits, but instead indicate signals of increased harm with respect to long-term mortality. All these observations and the concerns that have been published should lead to a reevaluation of the validity of the GLN hypothesis in critically ill patients and probably also to new recommendations for the practice guidelines developed by organizations such as the European Society for Clinical Nutrition and Metabolism, the American Society of Parenteral and Enteral Nutrition and the Canadian Practice Guidelines Committee [15].

### Strengths and weaknesses

The strengths of our meta-analysis include the use of several methods to reduce bias: a comprehensive search of the worldwide literature, including trials published in languages other than English; duplicate data abstraction; specific criteria for searching and analysis; and no industry funding. We also focused on clinically important primary outcomes. Furthermore, we created funnel plots for all primary and key secondary endpoints examined to look for possible publication bias associated with these endpoints.

In contrast, we are aware that our meta-analysis has several limitations. Among these are the limited number of larger trials [14, 27] and the small number of trials included in certain subgroup analyses. Owing to the heterogeneity of the included studies, the internal validity of our findings should be interpreted with caution. We also unfortunately could not perform subgroup analysis for all endpoints, owing to the limited number of trials in which the particular endpoints were examined. Another potential weakness of our review is that the studies included were published over the course of 2 decades. This may be relevant, as in a time-sequential analysis, Fadda and coworkers studied the effects of GLN supplementation over time. They showed that only trials performed before 2003 manifested a positive signal, whereas more recent trials failed to demonstrate any positive treatment effect [78]. Hence, it appears that only older, small, single-center trials of intravenous GLN, when meta-analyzed, showed a positive treatment effect.

## Conclusions

In this comprehensive systematic review, we demonstrate that enteral GLN supplementation given in conjunction with EN support does not confer significant reductions in hospital mortality among critically ill patients, including trauma patients. However, it may reduce hospital mortality in burn patients. No effects on infectious morbidity or ICU LOS were observed. Hospital LOS was significantly reduced in critically ill and burn patients but not in trauma patients. However, the results of our meta-analysis are based mainly on smaller, single-center studies, and two recent multicenter trials have suggested potential harm of GLN. Therefore, enteral GLN supplementation cannot be recommended for critically ill patients. In burn patients, larger studies are warranted, as our observations of a beneficial effect are based on a small number of patients. Such a trial is currently underway worldwide (citation: see Clinical trials.gov ID #NCT00985205).

## Key messages

- In critically ill patients, including trauma patients, supplemental enteral GLN does not decrease hospital mortality, infectious morbidity or ICU LOS.
- Supplemental enteral GLN does significantly reduce hospital mortality in burn patients; however, the relevant studies were small.
- Supplemental enteral GLN significantly shortens hospital LOS in critically ill and burn patients but not in critically ill trauma patients.
- Supplemental enteral GLN should not be given to critically ill patients or trauma patients, as its benefits are limited.
- Moreover, results are based mainly on single-center studies, and two recent multicenter trials have suggested potential harm of GLN.
- More data on enteral GLN supplementation are warranted in burn patients as present observations of a benefit are based on a small number of patients.

## Abbreviations

CDC: Centers for Disease Control and Prevention
EN: Enteral nutrition
GLN: Glutamine
ICU: Intensive care unit
ITT: Intention to treat
IV: Intravenous
LOS: Length of stay
M-H: Mantel-Haenszel
NA: Not applicable
NJ: Nasojejunal
PN: Parenteral nutrition
RCT: Randomized controlled trial
REDOXS: Reducing Deaths Due to Oxidative Stress Trial
RevMan: Review Manager software
RR: Risk ratio
SD: Standard deviation
TPN: Total parenteral nutrition
WMD: Weighted mean difference
